# Distinct Cerebellar Responses for Flexing, Extending and Stroking Tasks Using 7 T fMRI

**DOI:** 10.1007/s10548-025-01135-w

**Published:** 2025-10-14

**Authors:** Emma J. P. Brouwer, Nikos Priovoulos, Wietske van der Zwaag

**Affiliations:** 1 Spinoza Centre for Neuroimaging, KNAW, Amsterdam, The Netherlands; 2 Computational Cognitive Computational Neuroscience & Neuroimaging, Netherlands Institute for Neuroscience, Amsterdam, The Netherlands; 3 Oxford Centre for Integrative Neuroimaging, FMRIB, Nuffield Department of Clinical Neurosciences, University of Oxford, Oxford, UK

**Keywords:** Human Cerebellum, High-field fMRI, Cerebellar Surfaces, Somatotopy, Sensorimotor integration

## Abstract

The cerebellum plays a crucial role in the control of hand movements, enabling fine motor skills such as clapping and writing. Neurological diseases can affect the cerebellum, often leading to motor impairment. However, the cerebellar organisation of specific motor and sensory tasks in humans is under-explored in vivo compared to the neocortex, due to a lack of acquisition and analysis methods that effectively portray cerebellar activation in-vivo due to the cerebellum’s thin and highly-foliated cortex. In the neocortex, by comparison, response differences between distinct motor and sensory tasks have been reported, implying an extensive sensorimotor organisation. Here, we studied the cerebellar functional responses during three distinct tasks: flexing, extending and stroking of digits 1, 3 and 5 using B1-shimmed 7T functional MRI. We analysed the data in the standard 3D-functional space and the surface space, respecting the dense foliation of the cerebellum. All tasks elicited individual digit responses, engaging the cerebellar cortex in distinct ways: Digit extension yielded larger, more bilateral activation clusters and less distinct progressions of digit representations in comparison to flexing and stroking tasks. The stroking responses were found more medial in the anterior lobe than the flexing and extending clusters. The anterior lobe clusters were larger for the extending and flexing tasks than for the stroking task. These results imply that the cerebellum is engaged differently when tasks with differing sensory/motor components are performed and that these differences exist on a (sub)millimetre scale, akin to the mesoscale organisation in the cerebral cortex.

**Supplementary Information:**

The online version contains supplementary material available at 10.1007/s10548-025-01135-w.

## Introduction

Our interaction with the outside world is mediated through movement, including speech and body language; therefore, the sensorimotor network of the human brain is extremely important for our daily functioning. The cerebellum contains important nodes of the sensory and motor networks (Mottolese 2013; van der Zwaag et al. 2013; Wiestler et al. 2011; Ciapponi et al. 2023; Brouwer et al. 2024) which can be disturbed in multiple neurological diseases, often leading to motor impairment, for example in multiple sclerosis, coeliac disease and cerebellar ataxia (Weier et al. 2014; Stoodley and Schmahmann 2010; Schmahmann 2004). Notwithstanding the importance of the cerebellum for our daily functioning, its functional organisation is still underexplored in vivo.

Morphologically, the cerebellum is subdivided into ten different lobules with lobules I-V forming the anterior lobe and lobules VI-IX forming the posterior lobe. Most of the research in cerebellar fMRI uses these anatomical (lobular) boundaries to describe functional clusters (Grodd et al. 2001; Mottolese 2013; Wiestler et al. 2011). Such approaches are pragmatically abstract over the finer cortical branches of the cerebellum, since they fall below the typical fMRI acquisition and analysis resolution. Nevertheless, more recent research (Boillat et al. 2020; King et al. 2019; Xue et al. 2021) demonstrates that functional boundaries are not coherent with lobular boundaries in the human cerebellum as functional regions can span multiple lobules and finer organisations are found within the cerebellar lobules (Nettekoven et al. 2024). More specifically, within lobules IV/V/VI and VIII, two sets of functional somatotopic body maps have been found, each including an orderly somatotopy of hand, digits, lips, foot and tongue representations (Ai et al. 2013; Boillat et al. 2020; Grodd et al. 2001; van der Zwaag et al. 2013; Wiestler et al. 2011). Note that contrary to the cerebral homunculi, the homunculi in the cerebellum contain representations of the ipsilateral body parts. Within the cerebellar somatotopic body map, more detailed somatotopic organisations also exist. For instance, within the cerebellar hand area, a posterior-anterior (PA) gradient from thumb (D1) to little finger (D5) was found in the anterior cerebellar lobe using a sensory paradigm, as well as a less consistently organised somatotopic digit gradient in the posterior lobe (van der Zwaag et al. 2013). Interestingly, a medial-lateral (ML) spatial activation shift, where the D1 area was positioned more medial, was found in the anterior cerebellar lobe using a motor-sensory paradigm (Wiestler et al. 2011). In this 3 mm isotropic resolution 3 T study, the active patches responded to both sensory and motor stimuli.

In the neocortex, different motor tasks such as flexing and extending of digits result in distinct activation patterns (Huber et al. 2020; Schellekens et al. 2018). Specifically, a somatotopic organisation was found for a flexing paradigm, while no such organisation was found for an extending paradigm (Schellekens et al. 2018). In contrast, using different acquisition and analysis methods, Huber et al found ’extending’ somatotopic maps in a mirrored organisation with respect to ’flexing’ maps (Huber et al. 2020). Additionally, extension of digits has shown to result in larger activation clusters in the neocortex and is hypothesised to be less specific (Schellekens et al. 2018). Furthermore, a study measuring the force applied of separate digits during flexing and extending tasks showed that it is more difficult to move distinct fingers during an extending task compared to a flexing task (Yu et al. 2010). It is currently unclear however if distinct tasks such as flexing, extending and stroking result in distinct activation patterns in the human cerebellum, i.e. if the human cerebellum mirrors the functional organisation of the neocortex.

To investigate the activation patterns associated with distinct tasks in the human cerebellum, we require high spatial resolution due to the dense foliation of the cerebellar cortex. Ultra-high field fMRI is particularly suitable for acquiring high spatial resolution images, as it offers higher contrast to noise ratios (SNR/CNR) compared to lower field strengths (Pohmann et al. 2016). However, the shorter rf-wavelength at 7 T can result in destructive B1-interference within the human brain. Specifically, the cerebellum’s position within the skull, means that a low area of B1+ (and therefore low signal) falls inside the cerebellum with typical head coils. This greatly reduces cerebellar image quality, especially in the posterior lobe, where function is particularly underexplored (van der Zwaag et al. 2013; Wiestler et al. 2011). Parallel imaging (pTx) can help to reduce this loss of signal as it allows for participant-specific tuning of each transmit channel to improve the B1-field homogeneity in specific target ROIs, in a process known as B1-shimming (Van Den Bergen et al. 2007; Mao et al. 2006; Berrington et al. 2021). A similar approach has been used in neck imaging with a comparable hardware configuration (de Buck et al. 2023).

With increased spatial resolution, it becomes possible to associate functional responses to the corresponding cerebellar anatomical structures. This is important because the extensive cerebellar cortex (almost similar in size to the cerebral cortex (Sereno et al. 2020)) is highly foliated and much thinner than the cerebral cortex (Andersen et al. 1992). This foliation makes analysis of functional responses challenging in the 3D space, and likewise complicates the unfolding of the cerebellar cortex. Earlier cerebellar surface reconstruction approaches (Diedrichsen 2006) were designed for lower-resolution data and achieve readability and user friendliness at the cost of a significant deformation and smoothing over the details of the cerebellar cortex (Sereno et al. 2020). An individual cortical surface representation, derived from high-resolution anatomical data, may fit the complexity of the cerebellar surface better, thus providing a visual aid to analyse functional organisation (Boillat et al. 2020).

Here, we investigated the organisation of the cerebellar activation in the anterior and posterior lobes using three distinct tasks: flexing, extending and stroking of digits 1 (thumb), 3 (middle finger) and 5 (little finger). We leveraged the high SNR of fMRI at 7 T combined with B1-shimming and individual surface reconstructions to visualise the functional somatotopic responses.

## Methods

### Participants and MR Acquisitions

Nine right-handed participants (22-42 years, 7 Female) took part in the study. In accordance with the declaration of Helsinki, all participants provided written informed consent, and the study was approved by the local ethics committee. All MRI data were acquired using a Philips Achieva 7 T MRI scanner (Phillips, Best NL) equipped with an 8Tx/32Rx (Nova Medical, USA) whole-head coil. Second order $B_0$ shimming was applied over a whole brain ROI, using MRCodeTool (v1.5.7). A DREAM $B_1^+$ map was acquired along with a gradient echo (FOV=224x224x168$mm^3$, voxel size=3.5$mm^3$, TR/TE=6ms/3ms, flip $\hbox {angle=7}^{\circ }$) in which excitation pulses were applied through each channel separately in subsequent volumes (Adriany et al. 2005; Nehrke et al. 2014). From these, optimal phase modulations were calculated to minimise the $B_1^+$ variation over the cerebellum using the MRCodeTool (Tesla Dynamic Coils, Zaltbommel, NL). A 3D-EPI slab covering the cerebellum was used for all functional acquisitions with the following parameters: $\hbox {voxel size=1x1x1mm}^{3}$, FOV=182x60x186$mm^3$, $\hbox {TR}_{volume}$=3300ms, TR/TE=6.2ms/21ms, and $\hbox {flip angle=20}^{\circ }$ close to the Ernst Angle for cerebellar gray matter at 7T. SENSE undersampling was used in both phase-encoding directions to reduce the volume acquisition time (Poser et al. 2010): $\hbox {SENSE}_{y/z}$= 2.60/3.27, combined with partial-$\hbox {fourier}_{y/z}$=0.8/0.8. A whole-head MP2RAGE sequence (Marques et al. 2010) with the following parameters was used as anatomical reference: TR/TE=6.2ms/2.3ms, $\hbox {T}_{I1}$=800ms and $\hbox {T}_{I2}$=2700ms, $\hbox {TR}_{volume}$=5500ms, $\hbox {flip angle=7}^{\circ }{/5}^{\circ }$, FOV=230x230x185$mm^3$ and voxel size at least 0.8mm isotropic. Universal kt-point pulses (Gras et al. 2017) were used for the excitation pulses of the MP2RAGE, to improve flip angle homogeneity and contrast homogeneity over the cerebellum (Oliveira et al. 2021).

### Experimental Set-Up

**Fig. 1:**
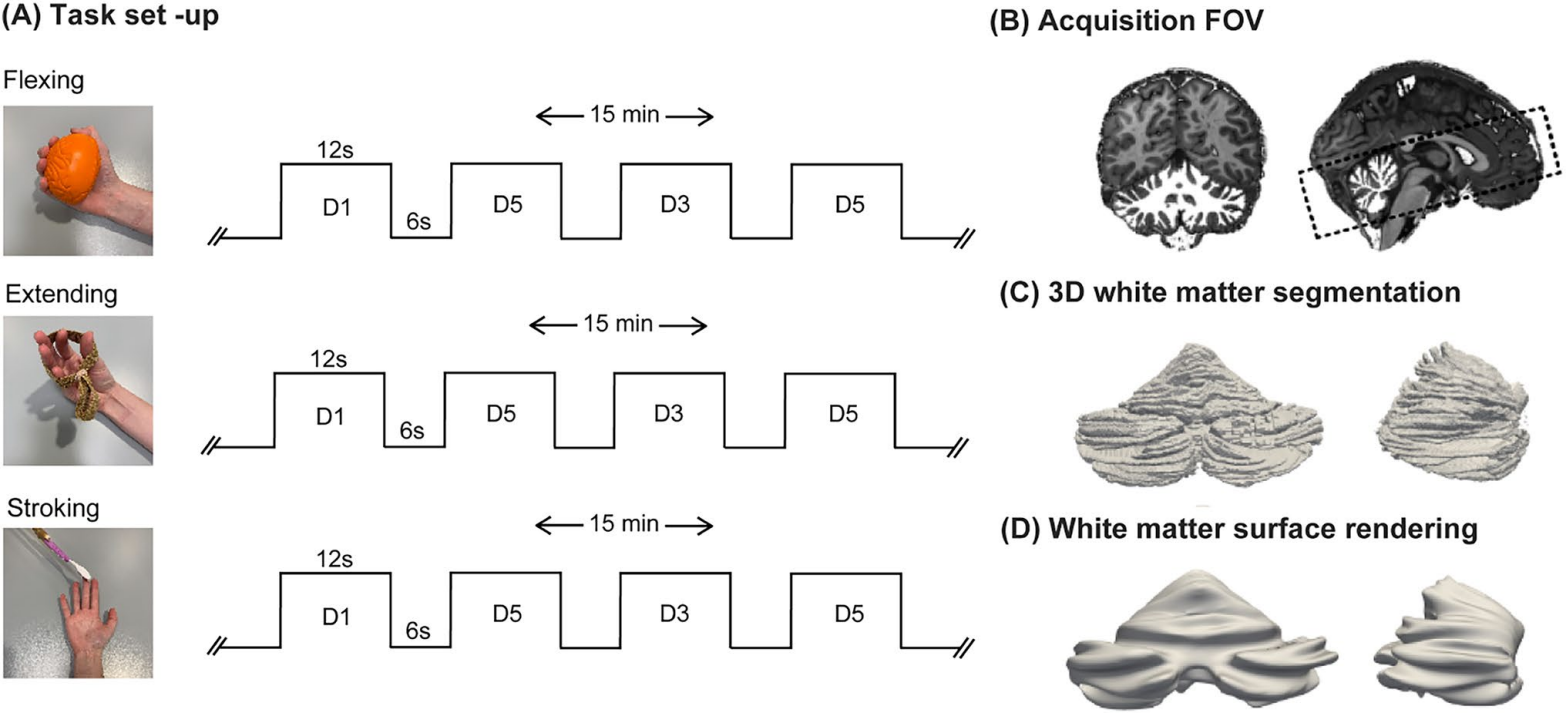
(**A**) Task timeline and experimental set up of the three tasks: flexing, extending and stroking. During the flexing and extending tasks the digit numbers were presented on a screen to the participants in the scanner. During the stroking task the auditory queue was only audible to the researcher. (**B**) Example 0.8mm isotropic MP2RAGE overlaid with the white matter mask of the cerebellar segmentation and a black box illustrating the EPI field of view. (**C**) A 3D surface rendering of the segmentation. (**D**) The resulting inflated surface

Each of the three functional tasks (flexing, extending and stroking) was performed during a separate 15-minute run with alternating 12 s ON and 6 s OFF blocks (Figure 1A). Stimuli (50 blocks per run) were applied to or executed with the right (dominant) hand. The participants practised the tasks outside of the scanner briefly before the session began to familiarise themselves with the pace of the tasks ($\approx$ 1 Hz) and to ensure they understood the visual cues. Participants were placed supine into the scanner, watching the screen through a rear-viewing mirror with their arms alongside their body in a manner that was comfortable for the participant. During the flexing task, the thumb (D1), middle finger (D3) or little finger (D5) was repeatedly flexed into a plastic ball, following visual cues showing a digit number: D1, D3 or D5 alternating with a cross:"+"to indicate blocks of rest, during which their hands were at rest. Visual cues were presented on an MR-compatible display (BOLD screen, CRS, UK). During the extending task, the participant extended the target digit against an elastic band following the same visual cues. For both flexing and extending tasks, digits were moved in a pseudo-random order, with an equal number of blocks for each digit (Figure 1A). During the stroking task, D1, D3 and D5 were sequentially stroked with a toothbrush (Figure 1A) by a researcher positioned next to the scanner bore, who received auditory cues (MRConfon, Cambridge, 2018). Visual and auditory cues were presented with Psychtoolbox scripts (Pelli 1997). The toothbrush used for the stroking task was attached to an MR-compatible stick such that the researcher did not have to lean into the scanner bore. To ensure consistency in the stroking frequency ($\approx$ 1 Hz), the same researcher performed the stroking stimulus during all sessions. After the scan session participants were asked how they experienced each task.

### Data Analysis

To retain spatial resolution, data were analysed where possible in the individual space, facilitated by the sensitivity of 7 T fMRI to the local BOLD signal changes (Cai et al. 2021; van der Zwaag et al. 2009). As specified below, we obtained max z-stats and ipsi/contralateral measures from the z-stats in functional space, the cerebellar cluster size and spatial distances from the individual anatomical space, we showed surface visualisation on the individual surfaces and finally we derived group spatial distances from data warped to MNI space.

#### Functional Space

The fMRI data were motion and distortion-corrected to the first volume using MCFLIRT and TOPUP (Andersson et al. 2003). A GLM (finger-stimulus>rest) was fitted (statistical significance: z=3.1, p<0.05, corrected for multiple comparisons using cluster-based thresholding, smoothing=1.5mm) using FSL FEAT (Jenkinson et al. 2012). For each of the three tasks, the significant cluster (p<0.05) for stimulation of each digit was identified both in the motor area of the posterior and anterior lobe of the ipsilateral hemisphere (King et al. 2019). Visual inspection confirmed that the activation pattern was accurately described by a single continuous cluster in each case. For each cluster, we inspected whether a contralateral cluster was present in addition to the ipsilateral one. The maximum z-stat value for each cluster was obtained in the posterior and anterior lobe per task and stimulated digit for all participants. A repeated measures ANOVA was performed to compare maximum z-stats across tasks, digits and lobes, followed by Post Hoc paired samples t-tests, Holm’s corrected ($p_{holm}<0.05$). All statistical analyses were performed using JASP (2022, Version 0.16.4.0).

#### Anatomical Space

fMRI data were registered to the individual anatomical space of the participant using linear registration (FLIRT)(Jenkinson et al., 2002). The cluster size was reported for the largest anterior and posterior lobe cluster in the cerebellar motor area. A repeated measures ANOVA was performed to compare cluster size across tasks, digits and lobules, followed by Post Hoc paired samples t-tests, Holm’s corrected ($p_{holm}<0.05$).

For each cluster, the weighted centre of gravity (wCOG), was calculated using a custom-written Matlab script (see supplementary materials) and the following equation:

Weighted centre of gravity

1 $$\begin{gathered} C_{x} = \frac{{\sum {(I(x,y,z) \cdot x)} }}{{\sum {I(x,y,z)} }},\quad C_{y} = \frac{{\sum {(I(x,y,z) \cdot y)} }}{{\sum {I(x,y,z)} }}, \hfill \\ C_{z} = \frac{{\sum {(I(x,y,z) \cdot z)} }}{{\sum {I(x,y,z)} }} \hfill \\ \end{gathered} $$

- $C_x, C_y, C_z$ are the coordinates of the weighted centre of gravity.
- *I*(*x*, *y*, *z*) represents the cluster z-stat (Z>3.1) at a given point (*x*, *y*, *z*).
- *x*, *y*, *z* are the voxel coordinates of each point in the cluster.

This equation ensures that voxel intensities (of the z stats) influence the location of the centre of gravity in space. In other words, if a voxel A has a higher z-score compared to a voxel B, the weighted centre of gravity between these two voxels will lie closer to voxel A than to voxel B. To assess task-related spatial shifts in digit representation locations, the Euclidean distance between the same digit wCOG was calculated pairwise between tasks (Flexing-Extending, Flexing-Stroking and Extending-Stroking). For distance calculations, significant activation was required for all three tasks and for all three digits. A repeated measures ANOVA was performed to compare differences in distance of task clusters of the same digit in Flexing-Extending, Flexing-Stroking and Extending-Stroking conditions, followed by Post Hoc paired samples t-tests, Holm’s corrected ($p_{holm}<0.05$).

#### Surface Space

To further present functional results on the continuous but highly foliated cerebellar cortical surface, individual-level surface reconstructions were derived. White matter and grey matter probability maps were extracted using CAT12. The SUIT toolbox cerebellar mask was used to limit the white and grey matter probability maps to the cerebellar area. The brain stem and dura were manually excluded using FSLeyes. The resulting white matter masks were upsampled by a factor of two, tessellated into a high-resolution mesh (Figure 1C) and inflated (Figure 1D) using the neuroimaging at high resolution Python package (Nighres) (Huntenburg et al. 2018). Only for visualisation purposes, a winner takes all (WTA) digit map was created in the anatomical space following van der Zwaag et al. (2013). The top 400 voxels for each digit were projected onto the inflated surface with a closest point approximation method using Nighres (Huntenburg et al. 2018). The closest point approximation can be chosen from the Nighres surface mapping function, which, in this context, means the 3D z-stat maps are projected onto the nearest point on the 2D cerebellar grey matter surface reconstruction. Paraview (Ahrens et al. 2005) was used to visualise the 3D surface maps and overlaying digit maps for the different tasks and participants.

#### MNI Space

Finally, to assess the directional consistency of possible spatial shifts between tasks in a shared anatomical reference space, the wCOGs were projected in MNI space for each task and lobe. This was achieved by registering individual MP2RAGEs to the 1 mm MNI space using nonlinear registration (FNIRT) (Andersson et al. 2007). Using the wCOG coordinates (obtained using Eq.1), The presence of a consistent orderly progression of digits was assessed by examining the sequential order of the wCOG coordinates for D1, D3, and D5 along the PA (Posterior-Anterior), ML (Medio-Lateral), and SI (Superior-Inferior) axes.

## Results

**Fig. 2:**
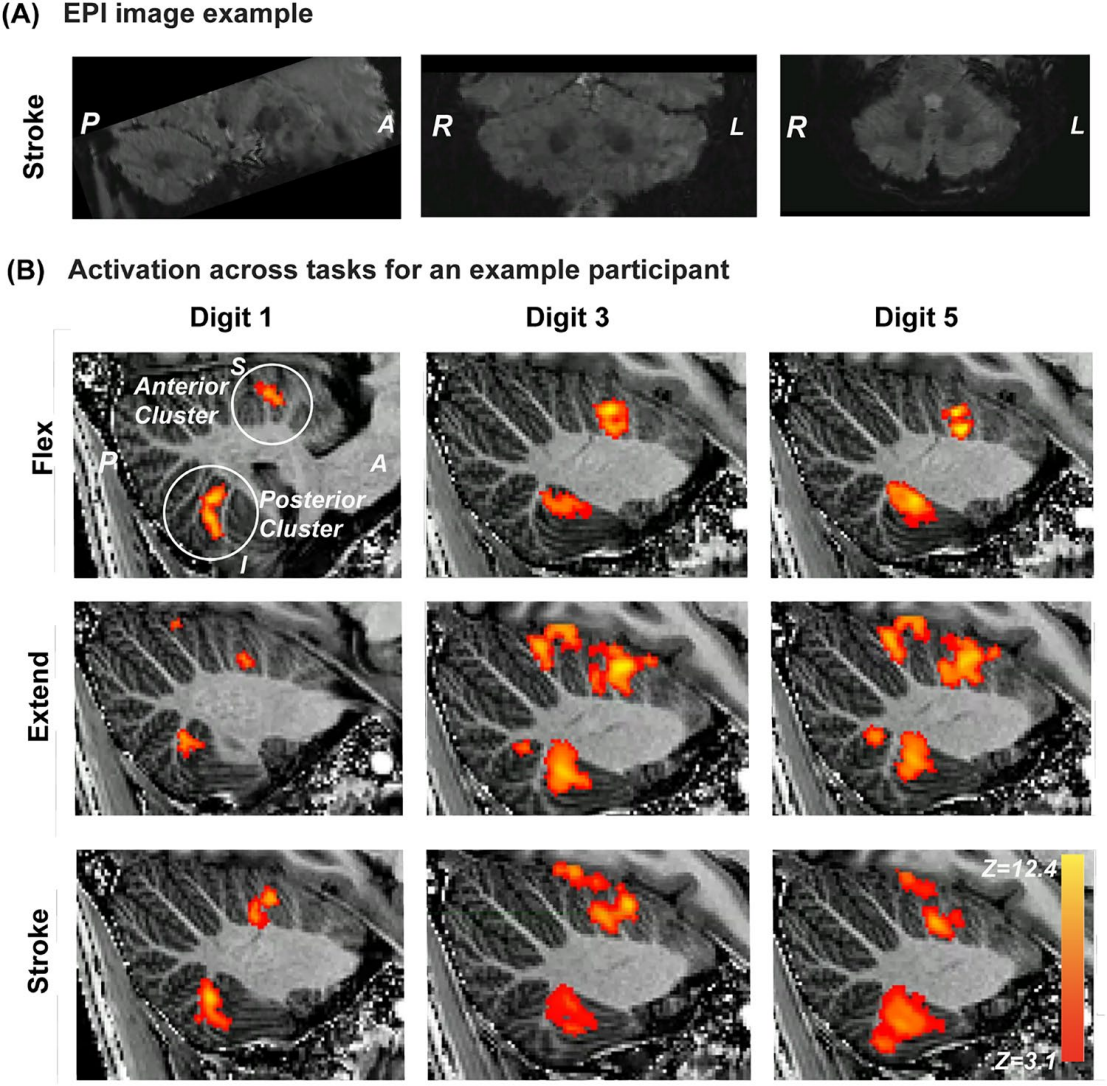
(**A**) EPI image example in the sagittal, coronal and axial view. (**B**) Thresholded z-stats for a single individual overlaid onto the MP2RAGE acquisition. Sagittal slices indicating activation across tasks (rows) and digits (columns). The activation cluster more superior is located in the anterior cerebellar lobe and the cluster more inferior is located in the posterior cerebellar lobe

An example EPI image from which BOLD responses were derived is shown in Figure 2A. Figure 2B displays activation across tasks for an example participant ($\#$1). Note the reliability of cerebellar BOLD responses to these tasks. Across participants we consistently found clusters in the posterior lobe (lobule VIII) and anterior lobe (lobule V) cerebellar motor area, with some clusters extending into lobule IV and VI. Across tasks (3), digits (3) and participants (9), 76 out of 81 clusters were detected in the anterior lobe and 71 out of 81 were detected in the posterior lobe. After the scan session, some participants reported that they found the extending task more difficult compared to the flexing and stroking task.

### Activation Clusters

**Fig. 3:**
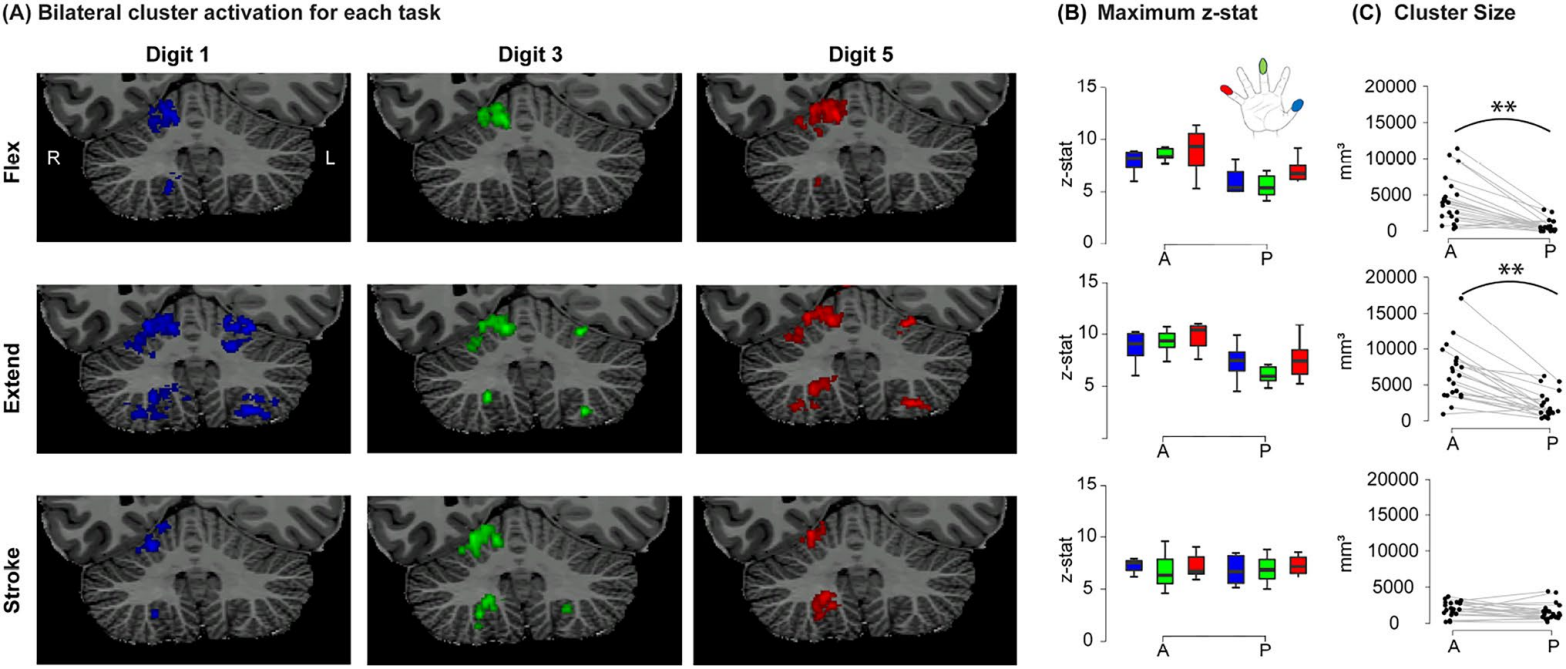
(**A**) The clusters for each digit and task overlaid on the coronal slice of the anatomy of an example participant. Note the consistent bilateral clusters presented in the extending task and lack thereof in the flexing and stroking tasks. (**B**) The maximum z-stat for each digit cluster across participants, tasks, digits and lobes. Note the higher z-stats in the anterior lobe during the flexing and extending tasks. (**C**) The number of voxels within a p<0.05 corrected cluster across participants, tasks and lobes. Note the larger clusters in the anterior lobe compared to the posterior lobe for the flexing and extending tasks. ** indicate significant differences (Post Hoc Holm’s corrected paired samples t-test p<0.01), between lobes across digits

All participants (9/9) showed bilateral responses during the extending tasks in both the anterior and posterior lobes (Figure 3A). In contrast, bilateral responses were inconsistent across participants during flexing and stroking tasks (4/9 and 6/9 respectively, Figure 3A). Flexing and extending task clusters had significantly higher z-stats in the anterior lobe compared to the posterior lobe (F(2,12)=15.30, p<0.001, supplementary tables 1-4). This was not the case for the stroking task, where z-stats were of similar strength in both lobes (Figure 3B, supplementary table 1).

Extending task clusters yielded larger clusters compared to flexing (Posthoc comparison: t(2)=2.88), p=0.023)) and stroking (t(2)=3.91,p=0.01) tasks (Figure 3C). In addition, clusters were larger in the anterior lobe compared to the posterior lobe specifically for extending and flexing tasks, but not for the stroking task (F(2,12)=13.01,p<0.001; supplementary tables 5-9), as illustrated in Figure 3C.

### Digit Progressions

**Fig. 4:**
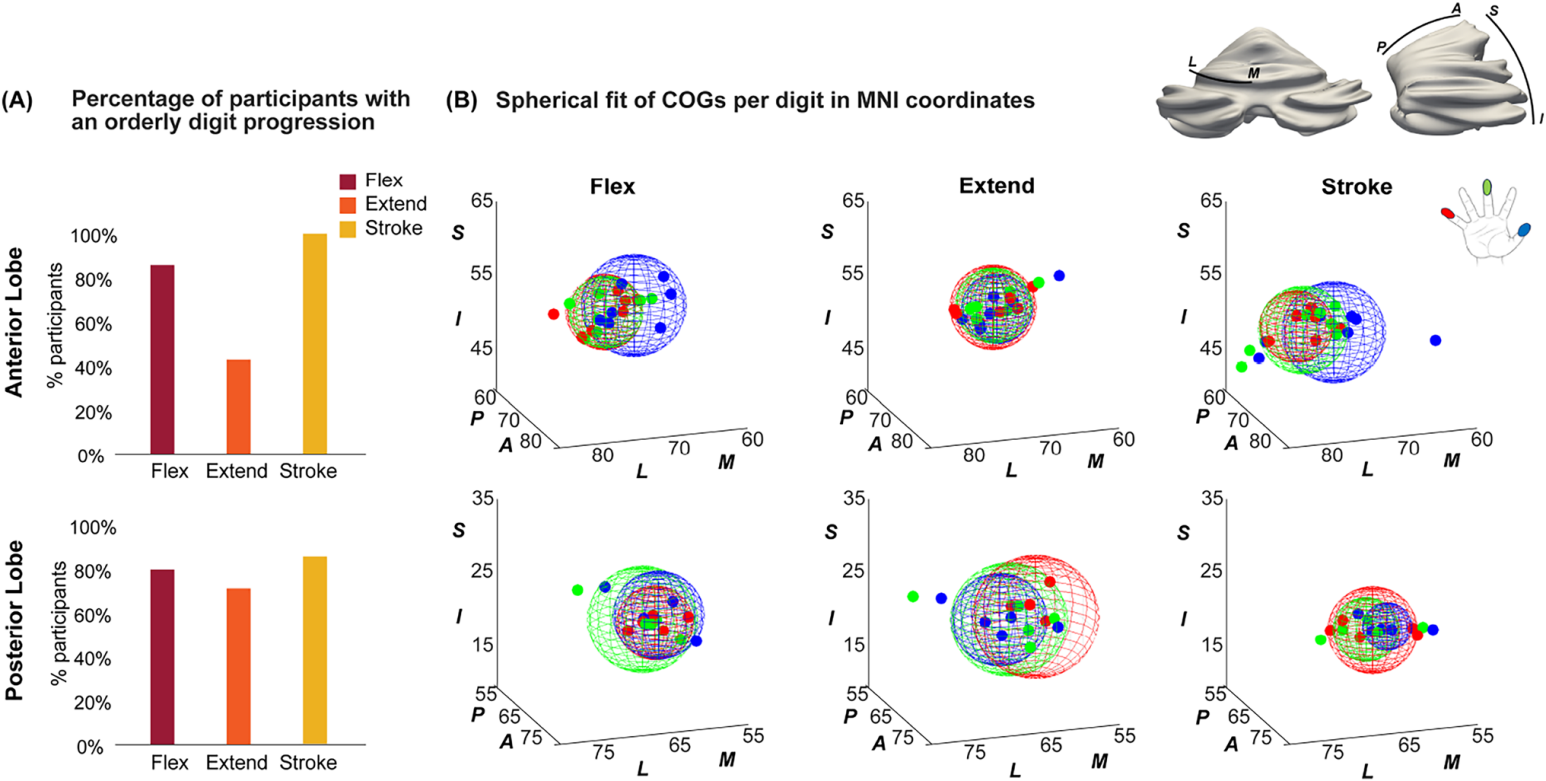
(**A**) The percentage of subjects with an orderly digit progression (Where the wCOG D3 was found between D1 and D5). (**B**) Plot of wCOGs of all three digits (D1, D3, D5) in MNI-space for each task including the group spherical fit to each task in the anterior lobe (top) and the posterior lobe (bottom). Note in the anterior lobe: the distance between the stroking task and the two motor tasks is larger compared to the distance between the two motor tasks for all digits. The flexing and extending tasks are found more superior and medial compared to the stroking tasks. In the posterior lobe spherical fits are close and overlapping

Consistent digit progressions were found in both lobes (Figure 4A). Figure 4B shows the MNI-space plot of the wCOGs for each digit group. In the anterior lobe, the flexing and stroking task responses both show a progression of digit representations where D1 is found more medially as well as posterior to D5. In the extending task, digit clusters were highly overlapping and progressions were not consistent in any direction. Although orderly progressions were found in the posterior lobe for more than half of participants (Figure 4A, supplementary table 10) across all tasks, these were inconsistent in their direction, resulting in overlapping plots in MNI space (Figure 4B, posterior).

### Spatial Shifts of Activation Clusters Between Tasks

**Fig. 5:**
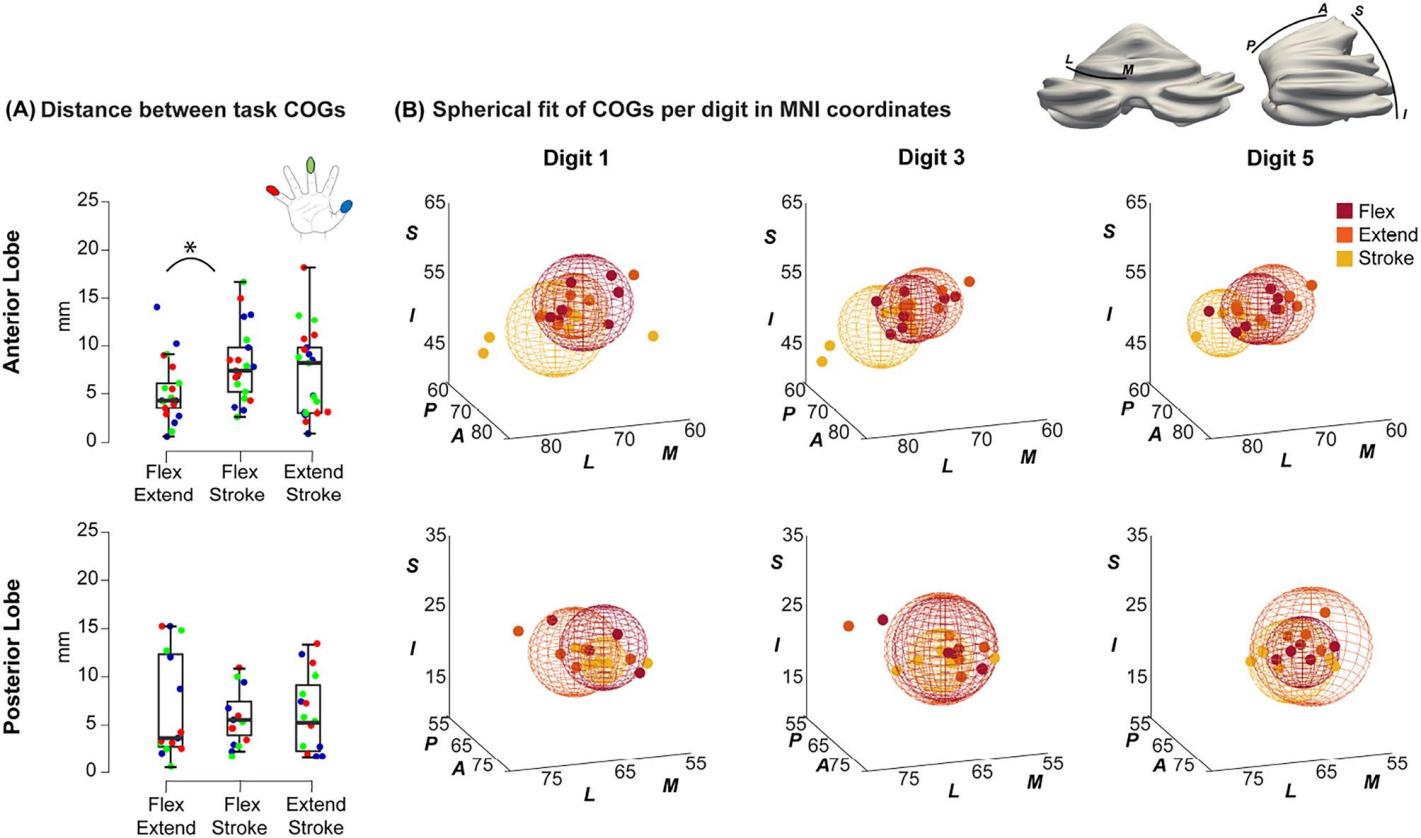
(**A**) The distances between task wCOGs for each digit. This was calculated for the participants own anatomical space. *Indicates significant differences (paired samples t-test $p_{holm}<$0.05). (**B**) The MNI-space COG plot of each digit including the mean spherical fit for each digit. Notice the thumb (blue sphere) is more posterior and medial in comparison to the little finger (red sphere) in both lobes in the flexing and the stroking task. The extending task shows largest overlap between digits in the anterior lobe. In the anterior lobe flexing and stroking spheres are more separated, showing a more distinct progression compared to the posterior lobe

**Fig. 6:**
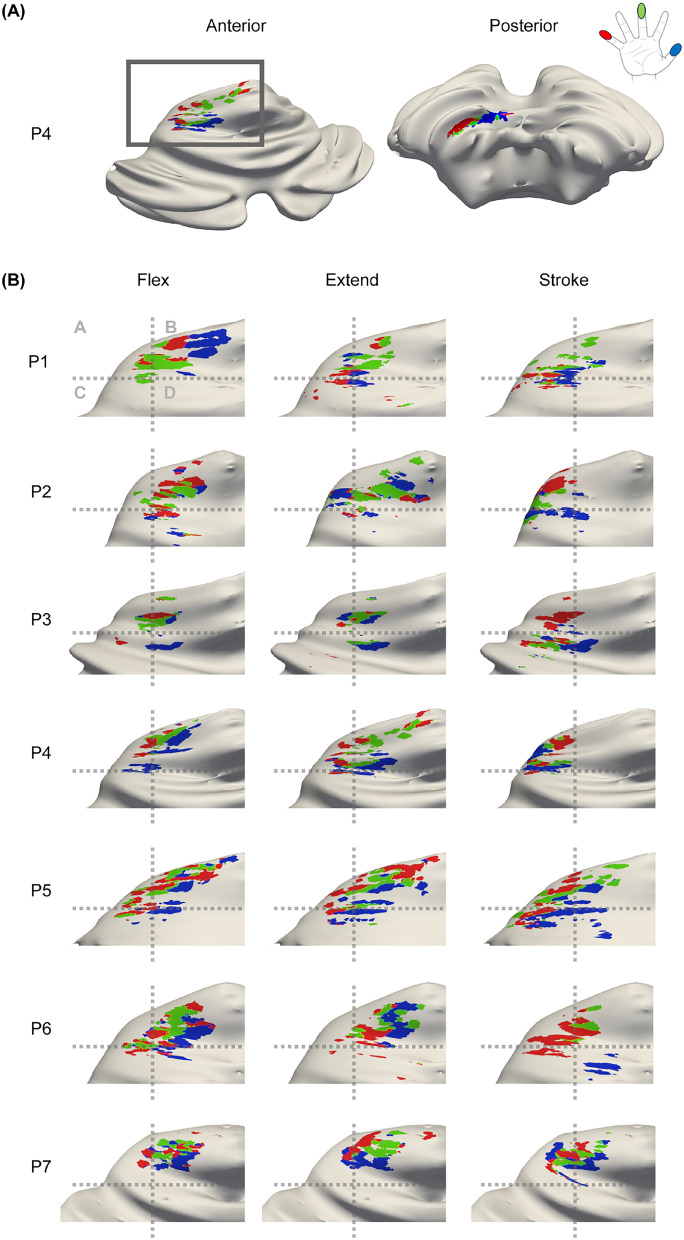
(**A**) The surface rendering of participant 4 from an anterior and posterior view during the extending task. (**B**) Activation of each task in the anterior lobe. Notice the consistent medio-lateral organisation of the digit clusters. The responses to the stroking task were located more laterally compared to the flexing and extending responses for all participants

A consistent mediolateral spatial shift was detected in the anterior lobe between the stroking task and the other two tasks. This is illustrated in Figure 5A, where the distances between task wCOGs for each lobe are presented. In the anterior lobe, the distances between task wCOGs for each digit were significantly larger between the flexing and stroking (M=8.0mm, SD=3.9mm) tasks compared to between the flexing and extending (M=5.2mm, SD=3.2mm) tasks (t(20)=−3.1,p=0.006) (supplementary tables 11-13). This is further visualised in Figure 5B in MNI coordinates (centre of sphere = mean wCOGs, sphere radius = distance standard deviation), where the stroking mean wCOG is visibly more lateral and inferior compared to the flexing and extending wCOGs in the anterior lobe across all digits. This difference is not observed in the posterior lobe, where flexing, extending and stroking wCOGs are close (non-significantly different distances in the individual anatomical space (Figure 5A) and overlapping spherical fits in MNI; Figure 5B). Additionally, we projected the WTA digit maps for each task on the participant-specific inflated corticocerebellar surface for visual inspection while accounting for the continuous cerebellar cortical surface (Figure 6). The flexing and extending task clusters were consistently found more medial compared to the stroking task clusters in the anterior cerebellum. This can be seen clearly in figure 6B: the stroking clusters are more prevalent in quadrants A and C, the flexing and extending clusters are prevalent in quadrant B and D (a fixed-position gridline is presented in grey to allow comparisons). In the posterior lobe, these task gradients were much less consistent and even though they were found for some participants, the direction of these gradients were not consistent across participants. This agrees with the visualisation of results in MNI space (Figure 4B). This is possibly due to the intense foliation in both ML and PA direction in the posterior lobe.

## Discussion

Cerebellar BOLD responses to flexing, extending and stroking tasks were found in both the anterior and posterior lobes of the cerebellum using 7 T fMRI with region-specific B1-shimming. Consistent individual digit representations were found in line with a map-like somatotopic topography of the cerebellar cortex. Nevertheless, cerebellar functional response patterns differed depending on the performed task as discussed in more detail below.

First of all, there were differences between the extending task compared to the flexing and stroking task. The extending task resulted in consistent bilateral activation in both lobes of the cerebellum whereas the flexing and stroking tasks did not. Previous studies on cerebellar activation during unilateral tasks have reported mixed findings. While some have not observed bilateral activation patterns during unilateral motor (Xue et al. 2021) or sensory (van der Zwaag et al. 2013) stimulation of the hand, others have documented bilateral activation e.g. in the context of pain-related stimuli (Misra and Coombes 2016). In the neocortex, bilateral activation in M1 scales with the difficulty of the task (Chettouf et al. 2020). The consistent bilateral activation during the extending task in our study may be driven by its task difficulty or salience, implying that the cerebellar cortex recruits neuronal populations across hemispheres to meet more complex manoeuvres. This agrees with the perceived extending task difficulty informally reported by the participants after the scan sessions and formally tested in a previous behavioural study (Yu et al. 2010).

In addition, and in contrast to the flexing and stroking experiments, we did not observe a consistent somatotopic organisation of the digit representations with the extending task. Earlier findings in the neocortex comparing flexing and extending tasks, show BOLD responses resulting from extending tasks are less digit-specific, likely due to receiving feedback from more digits, compared to flexing (Schellekens et al. 2018). In agreement with this idea, the extending clusters found in the current study were also larger compared to those of the flexing and stroking tasks. Note that the extension task may involve a different amount of proprioceptive feedback from the joints as they resist the force from the band. Nevertheless, in the neocortex opposite directions of somatotopic gradients between flexing and extending have been found using CBV-weighted imaging (Huber et al. 2020). The extending task used in the current study is highly similar to that used by Huber and colleagues and is therefore expected to yield similarly specific sensory input and require similar motor output as well. Hence, the lack of a somatotopic gradient in the human cerebellum for this extending task might suggest a different layout. Thereby, our findings provide a counterexample to the traditional closed-loop hypothesis of cerebellar cortical function (Kelly and Strick 2003), where one would expect a one-to-one representation between neocortical and cerebellocortical sensorimotor regions. Similar differences between neocortical and cerebellar functional convergence across tasks were recently reported (King et al. 2023; Brouwer et al. 2024). In addition, studies in rodents show a specific functional organisation inherent to the cerebellum (Aoki et al. 2019). Here, the cerebellar cortex is known to contain smaller functional units such as parasagittal stripes, which form a zonal organisation in the cerebellar cortex, where motor-sensory input from the spinal cord targets specific stripes, adjacent to those receiving descending feedback (Craciun et al. 2018; Pakan et al. 2007; Aoki et al. 2019). In humans, such a scale is currently not reached in functional imaging studies, but more recent research (Nettekoven et al. 2024) does show that finer functional organisations exist within the cerebellar motor areas. If in the future sufficiently high resolution diffusion tractography data becomes available, the parasagittal stripe pattern would be interesting to explore in the context of our functional MRI results.

Moreover, the stroking task elicited different responses compared to the flexing and extending tasks. The stroking task resulted in similar activation cluster sizes and z-stats in the posterior and anterior lobe of the cerebellum, whereas flexing and extending tasks engaged the anterior lobe more strongly than the posterior lobe. This pattern makes us confident that the observed differences between activation strength and cluster size are not due to anatomical or rf-coil sensitivity differences between the posterior and the anterior lobe in the cerebellum, but due to the task. We also found larger spatial distances between activation clusters of the stroking task and the corresponding clusters of the flexing and extending tasks. This could indicate a separation between motor and sensory organisation in the cerebellum, similar to the neocortex, where distinct sensory (S1) and motor digit maps (M1) exist (Huber et al. 2020; Wiestler et al. 2011). However, while we observe a spatial shift between these tasks, where the flexing and extending task contain more motor components and the stroking task more sensory, we cannot attribute this difference solely to the motor and sensory distinctions, as the flexing and extending task also create some sensory neuronal input (Brouwer et al. 2024). Similarly, the flexing and extending tasks are actively performed by the participant whereas the stroking task is not. As a result, the flexing and extending tasks involve a component of somatosensory reafference due to self-generated movement whereas the stroking task only involves afferent signalling.

Lastly, using the current experimental design, we cannot exclude the possibility that other digits were moving when the flexing and extending tasks were performed. However, since we find a consistent organisation in the flexing task and not for the extending task, we are suggesting that these differences are not fully driven by unintentional movement of non-target digits.

This study highlights the difficulties of analysing high-resolution BOLD activation data in the cerebellum, specifically in the posterior lobe. Across tasks, the posterior lobe presented a less consistent somatotopic organisation compared to the anterior lobe. This agrees with earlier sensory task fMRI results (van der Zwaag et al. 2013), where digit progressions were found in the posterior lobe, but in inconsistent directions across participants. In the earlier study, this variation might be explained by the orientation of the folds in the posterior lobe and their foliation both in a ML direction as well as in the AP direction. Hence, to avoid foliation effects in the current study, we used individual-specific cerebellar surface reconstructions, but the analysis in the posterior lobe would still benefit from higher anatomical fidelity surfaces, possibly through higher spatial resolution and analysis methods that better capture the underlying cerebellar structure (Priovoulos et al. 2023). Nevertheless, the cortex in the posterior lobe is known to be different from that in the anterior lobe in certain aspects that are functionally relevant (Jacobi et al. 2021; Pakan et al. 2007), and we cannot assume that the differences observed here are only due to the current hardware setup.

In summary, we reliably visualised the digit representation maps for flexing, extending and stroking tasks in the human cerebellar lobes using 7 T fMRI. The cerebellar cortex is differentially engaged depending on the sensorimotor action, with the extension task yielding larger, more bilateral, activation clusters and less distinct progressions of digit representations, possibly reflecting increased task difficulty. The stroking digit maps were separated in the medio-lateral direction from the flexing and extending maps, with the stroking maps found more lateral. This finding potentially aligns with the stripe and zonal functional organisation of the cerebellum identified in animal studies. The sensorimotor representation differences occur on a (sub)millimeter scale, similar to the mesoscale organisation observed in the cerebral cortex.

## Supplementary Information

Below is the link to the electronic supplementary material.

Supplementary file 1 (pdf 171 KB)

## Data Availability

The datasets generated and analyzed during the current study are not publicly available due to European regulations but are available from the corresponding author upon reasonable request.
