# Supplementary material for: Distinct Cerebellar Responses for Flexing, Extending and Stroking Tasks Using 7 T fMRI
Source: Brain Topogr. 2025 Oct 14;38(6):76. doi: 10.1007/s10548-025-01135-w (PMC12521328; doi:10.1007/s10548-025-01135-w)
Supplement: Supplementary file 1 — (pdf 171 KB) [file 10548_2025_1135_MOESM1_ESM.pdf]

5 Appendix

Table 1: **Descriptives maximum z-stats**

**Task**	**Digit**	**Lobe**	**N**	**Mean**	**SD**	**SE**	**COV**
Flex	D1	Posterior	7	5.286	2.609	0.986	0.493
		Anterior	7	8.200	1.557	0.588	0.190
	D3	Posterior	7	5.262	2.866	1.083	0.545
		Anterior	7	8.807	1.031	0.390	0.117
	D5	Posterior	7	6.135	2.900	1.096	0.473
		Anterior	7	8.892	2.400	0.907	0.270
Extend	D1	Posterior	7	7.343	1.752	0.662	0.239
		Anterior	7	8.748	1.541	0.582	0.176
	D3	Posterior	7	6.668	2.086	0.788	0.313
		Anterior	7	9.288	1.151	0.435	0.124
	D5	Posterior	7	7.554	1.954	0.739	0.259
		Anterior	7	9.767	1.367	0.517	0.140
Stroke	D1	Posterior	7	7.422	2.576	0.974	0.347
		Anterior	7	7.007	1.249	0.472	0.178
	D3	Posterior	7	7.287	2.211	0.836	0.303
		Anterior	7	6.774	1.825	0.690	0.269
	D5	Posterior	7	7.944	2.423	0.916	0.305
		Anterior	7	7.262	1.216	0.460	0.168

Table 2: **Within subject effects: maximun z-stats**

**Cases**	**Sum of Squares**	**df**	**Mean square**	**F**	**p**
Task	30.892	2	15.446	1.538	0.254
Residuals	120.544	12	10.045		
Digit	9.575	2	4.788	3.987	0.047*
Residuals	14.408	12	1.201		
Lobe	74.547	1	74.547	5.002	0.067
Residuals	89.422	6	14.904		
Task x Digit	1.012	4	0.253	0.170	0.951
Residuals	35.637	24	1.485		
Task x Lobe	72.985	2	36.492	15.300	<.001*
Residuals	28.621	12	2.385		
Digit x Lobe	1.972	2	0.986	1.142	0.352
Residuals	10.362	12	0.863		
Task x Digit x Lobe	2.050	4	0.512	0.751	0.567
Residuals	16.373	24	0.682		

Table 3: **Post Hoc Comparisons - Digit.**
P-values are adjusted using Holm's correction

		**Mean Difference**	**SE**	**t**	**pholm**
D1	D3	-0.013	0.239	-0.055	0.957
	D5	-0.591	0.239	-2.473	0.088
D3	D5	-0.578	0.239	-2.418	0.088

Table 4: **Post Hoc Comparisons - Task**
P-values are adjusted using Holm's correction

		**Mean Difference**	**SE**	**t**	**pholm**
Flex	Extend	-1.131	0.692	-1.635	0.384
	Stroke	-0.186	0.692	-0.268	0.793
Extend	Stroke	0.945	0.692	1.367	0.394

Table 5: **Descriptives cluster size**

**Task**	**Digit**	**Lobe**	**N**	**Mean # voxels**	**SD**	**SE**	**COV**
Flex	D1	Posterior	7	711.829	1020.575	385.741	1.434
		Anterior	7	3866.400	3381.512	1278.091	0.875
	D3	Posterior	7	436.529	451.504	170.653	1.034
		Anterior	7	3297.200	1562.616	590.613	0.474
	D5	Posterior	7	978.286	927.340	350.501	0.948
		Anterior	7	5467.586	4261.798	1610.808	0.779
Extend	D1	Posterior	7	2034.257	1911.209	722.369	0.940
		Anterior	7	5754.200	3230.208	1220.904	0.561
	D3	Posterior	7	1345.486	1023.549	386.865	0.761
		Anterior	7	5686.943	3077.539	1163.201	0.541
	D5	Posterior	7	3151.014	2050.332	774.953	0.651
		Anterior	7	8348.900	4737.536	1790.620	0.567
Stroke	D1	Posterior	7	1456.214	1457.832	551.009	1.001
		Anterior	7	1884.343	960.338	362.974	0.510
	D3	Posterior	7	1395.543	775.716	293.193	0.556
		Anterior	7	1954.700	1274.440	481.693	0.652
	D5	Posterior	7	1783.900	1244.340	470.316	0.698
		Anterior	7	2181.957	874.880	330.673	0.401

Table 6: **Within subjects effects: cluster size**

Cases	Sum of Squares	df	Mean square	F	p
TASK	1.540×10+8	2	7.698×10+7	8.218	0.006*
Residuals	1.124×10+8	12	9.367×10+6		
Digit	3.959×10+7	2	1.979×10+7	2.766	0.103
Residuals	8.587×10+7	12	7.156×10+6		
Lobe	2.460×10+8	1	2.460×10+8	19.285	0.005*
Residuals	7.653×10+7	6	1.275×10+7		
TASK x Digit	1.482×10+7	4	3.705×10+6	0.892	0.484
Residuals	9.964×10+7	24	4.152×10+6		
TASK x Lobe	9.012×10+7	2	4.506×10+7	13.005	<.001*
Residuals	4.158×10+7	12	3.465×10+6		
Digit x Lobe	5.193×10+6	2	2.597×10+6	1.113	0.360
Residuals	2.800×10+7	12	2.333×10+6		
TASK x Digit x Lobe	3.987×10+6	4	996645.017	0.654	0.630
Residuals	3.658×10+7	24	1.524×10+6		

Table 7: **Post hoc comparisons cluster size: task**
P-values are adjusted using Holm's correction

		Mean Diff	SE	t	p-holm
Flex	Extend	-1927.162	667.876	-2.886	0.027*
	Stroke	683.529	667.876	1.023	0.326
Extend	Stroke	2610.690	667.876	3.909	0.006*

Table 8: **Post hoc comparisons cluster size: lobe**

		Mean Diff	SE	t	p-holm
Anterior	Posterior	2794.352	636.314	4.391	0.005*

Table 9: **Post hoc comparisons cluster size: Task x Lobe**
P-values are adjusted using Holm's correction

		**Mean Diff**	**SE**	**t**	**pholm**
Flex, Anterior	Extend, Anterior	-2386.286	781.697	-3.053	0.063
	Stroke, Anterior	2203.395	781.697	2.819	0.096
	Flex, Posterior	3501.514	790.496	4.430	0.008*
	Extend, Posterior	2033.476	951.815	2.136	0.323
	Stroke, Posterior	2665.176	951.815	2.800	0.096
Extend, Anterior	Stroke, Anterior	4589.681	781.697	5.871	<.001*
	Flex, Posterior	5887.800	951.815	6.186	<.001*
	Extend, Posterior	4419.762	790.496	5.591	0.001*
	Stroke, Posterior	5051.462	951.815	5.307	<.001*
Stroke, Anterior	Flex, Posterior	1298.119	951.815	1.364	0.945
	Extend, Posterior	-169.919	951.815	-0.179	1.000
	Stroke, Posterior	461.781	790.496	0.584	1.000
Flex, Posterior	Extend, Posterior	-1468.038	781.697	-1.878	0.451
	Stroke, Posterior	-836.338	781.697	-1.070	1.000
Extend, Posterior	Stroke, Posterior	631.700	781.697	0.808	1.000

Table 10: **Spherical fit in MNI space coordinates**

Task, Lobe	Digit	Radius (mm)	X (ML)	Y (PA)	Z(SI)
Flex, Anterior	D1	7.13	69.24	71.68	54.13
	D3	4.49	73.25	71.59	52.94
	D5	4.46	73.48	70.74	52.83
Extend, Anterior	D1	4.69	69.98	69.79	53.38
	D3	4.72	71.03	70.82	53.52
	D5	4.12	69.58	68.91	52.65
Stroke, Anterior	D1	5.51	70.98	68.06	49.24
	D3	5.29	76.01	66.79	49.46
	D5	4.64	77.4	65.56	49.74
Flex, Posterior	D1	5.76	65.22	74.49	24.27
	D3	6.96	66.34	71.46	22.91
	D5	4.85	65.27	73.27	22.87
Extend, Posterior	D1	6.05	67.76	74.49	24.27
	D3	7.39	66.83	70.97	22.78
	D5	8.02	64.86	75.64	24.34
Stroke, Posterior	D1	3.14	65.13	74.09	22.35
	D3	4.20	67.31	72.29	21.61
	D5	5.69	66.31	71.84	21.30

Table 11: **Descriptives: distance between tasks anterior lobe**

Task pair	N	Mean (mm)	SD	SE	COV
Flex - Extend	21	5.238	3.246	0.708	0.620
Flex - Stroke	21	8.033	3.886	0.848	0.484
Extend - Stroke	21	7.214	4.538	0.990	0.629

Table 12: **Post hoc comparisons distance between tasks anterior lobe**
P-values are adjusted using Holm correction

		Mean Diff	SE	t	pholm
Flex - Extend	Flex - Stroke	-2.795	1.014	-2.756	0.026*
Flex - Extend	Extend - Stroke	-1.976	1.014	-1.948	0.117
Flex - Stroke	Extend - Stroke	0.819	1.014	0.808	0.424

Table 13: **Descriptives: distance between tasks posterior lobe**

**Task pair**	**N**	**Mean**	**SD**	**SE**	**COV**
Flex - Extend	15	6.633	5.522	1.426	0.833
Flex - Stroke	15	5.627	2.708	0.699	0.481
Extend - Stroke	15	5.920	4.161	1.074	0.703

Table 14: **Post hoc comparisons distance between tasks posterior lobe**
P-values are adjusted using Holm's correction

		**Mean Diff**	**SE**	**t**	**pholm**
Flex - Extend	Flex - Stroke	1.007	1.383	0.728	1.00
Flex - Extend	Extend - Stroke	0.713	1.280	0.557	1.00
Flex - Stroke	Extend - Stroke	-0.293	1.075	-0.273	1.00

The Matlab code to calculate the weighted centre of gravity.

```
% Define regions of interest
D1 = niftiread(["image path digit 1" ]);
D3 = niftiread(["image path digit 3" ]);
D5 = niftiread(["image path digit 5" ]);
%Take digit cluster images and concatenate them into a 4D matrix
task_stats(:,:,:,1) = D1 ;
task_stats(:,:,:,2) = D3 ;
task_stats(:,:,:,3) = D5 ;
%Calculate the COG using the regionprops3 function.
% Define array with the COGs for D1 D3 D5 showing y,x,z in each mask
cog_flex = [] ;
%Loop across the digits the task was performed with
for digit = [1 2 3]
%Create a grayscale image (otherwise the function does not work)
grayImage = task_stats(:,:,:,digit)/(max(task_stats(:,:,:,digit),[],'All'));
%Create a binary mask with single values to mask the area where the COG should be calculated
binaryImage = imbinarize(task_stats(:,:,:,digit));
labeledImage = single(binaryImage);
%Calculate the weighted centre of gravity
measurements_point = regionprops3(labeledImage, grayImage,'WeightedCentroid');
% put results in a single matrix for each digit
cog_flex = cat(3,cog_flex, table2array(measurements_point));
end
```
